# Antibacterial, Antiradical Potential and Phenolic Compounds of Thirty-One Polish Mushrooms

**DOI:** 10.1371/journal.pone.0140355

**Published:** 2015-10-15

**Authors:** Natalia Nowacka, Renata Nowak, Marta Drozd, Marta Olech, Renata Los, Anna Malm

**Affiliations:** 1 Chair and Department of Pharmaceutical Botany, Medical University of Lublin, Lublin, Poland; 2 Department of Pharmaceutical Microbiology, Medical University of Lublin, Lublin, Poland; National University of Ireland - Galway, IRELAND

## Abstract

**Background:**

Among many sources of natural bioactive substances, mushrooms constitute a huge and almost unexplored group. Fungal compounds have been repeatedly reported to exert biological effects which have prompted their use in pharmaceutical and cosmetic industry. Therefore, the aim of this study was analysis of chemical composition and biological activity of 31 wild growing mushroom species (including saprophytic and parasitic) from Poland.

**Methods:**

Qualitative and quantitative LC-ESI-MS/MS analysis of fourteen phenolic acids in the mushrooms analysed was performed. Moreover, total phenolic content was determined by the modified Folin-Ciocalteau method. Antioxidative activity of ethanolic extracts towards DPPH^•^ free radical was examined. Antibacterial activity against Gram-positive (*S*. *epidermidis*, *S*. *aureus*, *B*. *subtilis*, *M*. *luteus*) and Gram-negative (*E*. *coli*, *K*. *pneumoniae*, *P*. *aeruginosa*, *P*. *mirabilis*) microbial strains was analyzed.

**Results:**

As a result, the first such broad report on polyphenolic composition, antiradical and antimicrobial potential of wild growing Polish mushrooms was developed. Mushroom extracts were found to contain both benzoic (protocatechuic, 4-OH-benzoic, vanillic, syringic) and cinnamic acid derivatives (caffeic, *p*-coumaric, ferulic). Total phenolic content in mushrooms ranged between 2.79 and 53.13 mg gallic acid equivalent /g of dried extract in *Trichaptum fuscoviolaceum* and *Fomes fomentarius*, respectively. Fungi showed much differentiated antiradical activity, from highly active *F*. *fomentarius* to poorly effective *Russula fragilis* (IC_50_ 1.39 to 120.54 mg per mg DPPH^•^, respectively). A quite considerable relationship between phenolic content and antiradical activity has been demonstrated. Mushrooms varied widely in antimicrobial potential (MIC from 0.156 to 5 mg/ml). Generally, a slightly higher activity against Gram-positive than Gram-negative strains was observed. This is the first study concerning the chemical composition and biological activity of the majority of investigated species.

## Introduction

Since ancient times, mushrooms have been valued as both food and medicines. Medicinal mushrooms possess a long history of use, especially in Asian countries. However they have also played a crucial role in treatment of several diseases affecting rural populations of Eastern European countries. They have been used by preparing hot water extracts, concentrates or in powdered forms [1]. Species like *Inonotus obliquus*, *Fomitopsis officinalis*, *Piptoporus betulinus* and *Fomes fomentarius* have been found as useful agents in the treatment of gastrointestinal disorders, bronchial asthma and different types of cancers [2]. Moreover, bioactive compounds from mushrooms have been reported to exert immunomodulating, antiviral, antidiabetic, antitumor, antioxidant, radical scavenging and antibacterial effects [1, 3, 4]. Searching for natural constituents with such properties is highly desirable nowadays when the burden of civilization diseases (including cancers) affects all of humankind. There is also a growing interest in investigations of natural chemopreventive agents, which can act as blockers and/or suppressors by inhibiting carcinogenesis at the initiation, post-initiation as well as promotion stages.

Since antimicrobial resistance has spread around the world, the development of new drugs or searching for natural products supporting antibiotics is also necessary. The World Health Organization introduced the global report which indicates that antibacterial resistance threatens the prevention and treatment of various infections induced by microorganisms. *Staphylococcus aureus*, *Escherichia coli* or *Klebsiella pneumoniae* are among the most resistant strains posing a real risk to the society [5, 6]. Numerous diseases, even those that were once easily healed, are becoming a huge problem. The latest research revealed that uncultured bacteria constitute a source of new antibiotics. Ling et al. (2015) developed some methods to grow uncultured bacteria and they report new antibiotic, teixobactin without detectable resistance. Therefore, it is likely that some natural compounds with similarly low susceptibility to resistance occur in nature and searching for them is required [7].

Among many sources of natural bioactive substances, mushrooms constitute a huge and almost unexplored group. The number of mushroom species all over the world is estimated at about 150 000. However only 22 000 species are known to science and only approximately 5% of them has been studied [8]. Considering that many lower and higher fungi are already a valued source of active ingredients, we wanted to assess biological potential and chemical composition of 31 mushroom species growing wild in Poland. In this group, species with great therapeutic potential, defined in Polish literature as non-edible or medicinal can be found [9]. Some of them are saprophytic, growing from dead wood (*Bjerkandera adusta*, *Clavicorona pyxidata*, *Daedaleopsis confragosa*, *Gymnopilus penetrans*, *Hyphodontia paradoxa*, *Lenzites betulinus*, *Panellus stypticus*, *Psilocybe fascicularis*, *Psilocybe lateritia*, *Stereum hirsutum*, *Trametes hirsute*, *Trichaptum fuscoviolaceum*) or parasitic (fungal plant pathogens; *Fomes fomentarius*, *Fomitopsis pinicola*, *Heterobasidion annosum*). Majority of selected species provides huge mass of fruiting bodies which could be obtained in industrial amounts constituting interesting material for preparation of pharmaceutical and cosmetic products [2]. Therefore, the first aim of the present study was determination of antiradical activity of ethanolic extracts prepared from the 31 mushrooms species. We have decided to investigate the content of phenolic compounds (phenolic acids, in particular) in these fungi, as polyphenols belong to the best free radical scavengers [10]. Another task was to determine the effect of polyphenols on the mushrooms’ activity. There is a vast body of evidence suggesting that mushroom extracts exert antimicrobial potential [11]. Our previous study has also revealed the antibacterial activity of 19 edible fungi species [4]. Thus, we have decided that another direction of our study will be investigation of antimicrobial potential of aforementioned 31 extracts.

## Materials and Methods

### Materials

The wild growing fruiting bodies of 31 mushrooms—*Amanita citrina* (Schaeff.) Pers., *Amanita muscaria* (L.: Fr.) Hook., *Amanita pantherina* (DC.: Fr.) Krombh., *Amanita porphyria* (‘porphyrea’) (Alb. &Schwein.: Fr.) Mlady, *Bjerkandera adusta* (Willd.: Fr.) P. Karst., *Clavicorona pyxidata* (Pers.: Fr.) Doty, *Cortinarius armillatus* (Fr.: Fr.) Fr., *Cortinarius sanguineus* (Wulf.: Fr.) Fr., *Daedaleopsis confragosa* (Bolt.: Fr.) J. Schröt., *Fomes fomentarius* (L.: Fr.) Kickx, *Fomitopsis pinicola* (Swartz.: Fr.) P. Karst., *Gymnopilus penetrans* (Fr.: Fr.) Murrill, *Heterobasidion annosum* (Fr.) Bref. ss. Lato, *Hygrophoropsis aurantiaca* (Wulf.: Fr.) J. Schröt., *Hyphodontia paradoxa* (Schrad.: Fr.) E. Langer & Vesterholt ss. Lato, *Lactarius aurantiacus* (Pers.: Fr.) Gray, *Lactarius helvus* (Fr.) Fr., *Lactarius vellereus* (Fr.) Fr., *Lenzites betulinus* (‘betulina’) (L.: Fr.) Fr., *Panellus stypticus* (‘stipticus’) (Bull.: Fr.) P. Karst., *Pseudoclitocybe cyanthiformis* (Bull.: Fr.) Singer, *Psilocybe fascicularis* (Huds.: Fr.) Noordel., *Psilocybe lateritia* (Schaeff.: Fr.) Noordel., *Rhodocollybia maculata* (Alb. & Schwein.: Fr.) Singer, *Russula fragilis* (Pers.: Fr.) Fr. var fragilis, *Scleroderma citrinum* Pers., *Stereum hirsutum* (Willd.: Fr.) Gray, *Thelephora terrestris* Ehr. Ex Willd.: Fr., *Trametes hirsuta* (Wulf.: Fr.) Pilát, *Trichaptum fuscoviolaceum* (Ehrenb.: Fr.) Ryvarden, *Tubaria furfuracea* (Pers.: Fr.) Gillet, were collected in the Forests of Włodawa (Lublin Province, Poland; GPS coordinates for centre of the collection area: 51°31'1.33" N, 23°22'12.60" E) between 2012 and 2013. No specific permissions were required for these locations since collecting mushrooms in the Polish state forests is legally permitted. The field studies did not involve endangered or protected species according to the previous and current Regulation of Ministry of the Environment of Republic of Poland (Journal of Law 2004, No. 168, item 1765; Journal of Law 2014, item 1408). Mushroom specimens were authenticated by Dr. Zofia Flisińska from the Department of Botany and Mycology, Maria Curie-Skłodowska University, Lublin, Poland and deposited at the Department of Pharmaceutical Botany, Medical University of Lublin, Poland (voucher specimens: No. MSH-001, No. MSH-002, No. MSH-003, No. MSH-004, No. MSH-006, No. MSH-009, No. MSH-015, No. MSH-019, No. MSH-017, No. MSH-020, No. MSH-021, No. MSH-023, No. MSH-024, No. MSH-025, No. MSH-061, No. MSH-032, No. MSH-033, No. MSH-035, No. MSH-072, No. MSH-050, No. MSH-053, No. MSH-027, No. MSH-028, No. MSH-013, No. MSH-060, No. MSH-062, No. MSH-063, No. MSH-066, No. MSH-067, No. MSH-069, No. MSH-070, respectively). Mushrooms were immediately lyophilized in the Free Zone 1 apparatus (Labconco, Kansas City, KS, USA) and kept in a freezer until further analysis.

### Chemicals

Standards of gallic, protocatechuic, gentisic, 4-OH-benzoic, vanillic, caffeic, syringic, p-coumaric, ferulic, salicylic, veratric, synapic, 3-OH-cinnamic, rosmarinic acid and Trolox, 2,2-diphenyl-1-picrylhydrazyl radical (DPPH^•^), ascorbic acid were purchased from Sigma–Aldrich Fine Chemicals (St. Louis, MO, USA). Ethanol, methanol and the Folin–Ciocalteu reagent were from POCH (Gliwice, Poland). All the chemicals were of analytical grade. LC grade methanol (MeOH) was purchased from J.T. Baker (Phillipsburg, USA). LC grade water was prepared using a Millipore Direct-Q3 purification system (Bedford, MA, USA). All colorimetric measurements were conducted on 96-well transparent microplates (Nunclon, Nunc, Roskilde, Denmark) using an Elisa Reader Infinite Pro 200F (Tecan Group Ltd., Männedorf, Switzerland).

### Preparation of extracts

5 g of lyophilized and milled mushrooms were macerated twice with 50 ml of 99.8% ethanol at room temperature for 24 h. The suspensions were filtered through filter paper. The residues were twice extracted by ultrasonically assisted extraction with 50 ml of ethanol at room temperature for 30 minutes. The combined extracts were evaporated to dryness under vacuum. Samples were re-dissolved in the appropriate solvents for each determination.

### Total phenolic content (TPC)

Total phenolic content was assayed on microplates by the modified Folin-Ciocalteau method [12]. Briefly, 20 μl of the examined extract was added to 20 μl of diluted Folin-Ciocalteu reagent (with water 1:4, v/v), followed by addition of 160 μl of sodium carbonate (75 g/l). The absorbance was measured at 680 nm after 20 min using an Elisa reader with the solution containing water instead of the Folin-Ciocalteau reagent as a blank. The results were calculated on the basis of the standard curve prepared in the same conditions for gallic acid (curve equation: y = 0.0047x + 0.0227; r^2^ = 0.9989; linearity range 20–200 μg/ml) and expressed as mg of gallic acid per g of extract.

### LC-ESI-MS/MS conditions of analysis of phenolic acids

Phenolic acids contents were determined by reversed-phase high-performance liquid chromatography and electrospray ionization mass spectrometry (LC-ESI-MS/MS). For this purpose, an Agilent 1200 Series HPLC system (Agilent Technologies, USA) equipped with a binary gradient solvent pump, a degasser, an autosampler and column oven connected to 3200 QTRAP Mass spectrometer (AB Sciex, USA) was used. Chromatographic separations were carried out at 25°C, on a Zorbax SB-C18 column (2.1 x 50 mm, 1.8-μm particle size; Agilent Technologies, USA) with a mobile phase consisting of water containing 0.1% HCOOH (solvent A) and methanol containing 0.1% HCOOH (solvent B), using 3 μl injections. The flow rate was 500 μl/min and the gradient was as follows: 0–0.8 min– 5% B; 2–3 min– 20% B; 5–7.5 min– 100% B; 8.5–11 min– 5% B.

The QTRAP-MS system was equipped with electrospray ionisation source (ESI) operated in the negative-ion mode. ESI worked at the following conditions: capillary temperature 600°C, curtain gas at 25 psi, nebulizer gas at 60 psi, negative ionisation mode source voltage − 4500 V. Nitrogen was used as curtain and collision gas. For each compound, the optimum conditions of Multiple Reaction Mode (MRM) were determined. The data was acquired and processed using Analyst 1.5 software (AB Sciex, USA). The analytes were identified by comparing the retention time and m/z values obtained by MS and MS2 with the mass spectra from corresponding standards tested under the same conditions. The calibration curves obtained in MRM mode were used for quantification of all analytes.

The limits of detection (LOD) and quantification (LOQ) for phenolic compounds were determined at a signal-to-noise ratio of 3:1 and 10:1, respectively, by injecting a series of dilute solutions of known concentrations.

### DPPH^●^ assay

The scavenging effect of extracts was monitored as previously described with some modifications [12]. Aliquots of 180 μl of a freshly prepared 2,2-diphenyl-1-picrylhydrazyl (DPPH^●^) coloured solution in methanol (0.07 mg/ml) were mixed with 20 μl of the extract diluted to various concentrations in 96-well microplates. The solutions were shaken and incubated at 28°C for 60 min in the dark. A decrease in DPPH^●^ absorbance induced by the sample was measured at 517 nm using an Elisa reader.

Antiradical activity of extracts was calculated according to the following formula:

% Inhibition=[(AB−AA)/AB ]×100

Where: AB—absorption of a blank sample (DPPH^●^ solution and methanol instead of the test extract), AA—absorption of a tested sample with DPPH^●^ reagent.

A dose response curve for five prepared dilutions of each extract was plotted to determine the IC_50_ values. All tests were carried out in triplicate and averaged. Results were expressed as standard equivalents using Trolox (TE) and ascorbic acid (VCE) based on their IC_50_ values. Moreover, the antiradical efficiency (AE = 1/IC_50_) was calculated.

### Antibacterial assay *in vitro*

The antibacterial potential of extracts was evaluated using the micro-broth dilution method, which enables determination of the minimal inhibitory concentration (MIC) according to the early described procedure [13]. The eight reference strains, including Gram-positive bacteria (*Staphylococcus epidermidis* ATCC 12228, *Staphylococcus aureus* ATCC 25923, *Bacillus subtilis* ATCC 6633, *Micrococcus luteus* ATCC 10240) and Gram-negative bacteria (*Escherichia coli* ATCC 25922, *Klebsiella pneumoniae* ATCC 13883, *Pseudomonas aeruginosa* ATCC 9027, *Proteus mirabilis* ATCC 12453) were used. Extracts were dissolved in dimethyl sulfide (DMSO) and the series of the two-fold dilutions, ranging from 0.078 to 5 mg/ml, was prepared in Mueller-Hinton broth (Biocorp, Poland) in 96-well microtiter plates. The wells were inoculated with the bacterial suspension (the final inoculum size of 10^6^ colony-forming units—CFU/ml). Following the 24-hour incubation at 35°C, MIC was defined as the lowest concentration of the extract at which no visible growth was observed. DMSO was used as a negative control, while as a positive control only plant material in broth and broth with inocula was included. Gentamicin was used as a reference compound. Minimal bactericidal concentrations (MBCs) were determined by collecting 20 μl from each well with growth inhibition and placing onto duplicate Mueller-Hinton agar plates and incubating at 35°C for 24 h. MBC was defined as the lowest extract concentration at which there was no bacterial growth.

## Results and Discussion

### Total phenolic content (TPC)

Since polyphenols are considered strong antioxidants, the total phenolic content (TPC) was determined in all species [14]. For this purpose, the Folin–Ciocalteu’s assay was used. The results expressed as mg of gallic acid equivalents (GAE) per g of dried extract are presented in Table 1. TPC in the investigated mushrooms ranged between 2.79 and 53.13 mg GAE/g in *T*. *fuscoviolaceum* and *F*. *fomentarius*, respectively and was much higher than the results obtained for edible mushroom species reported in our previous paper [4]. Relatively high amounts were demonstrated for *A*. *citrina* (38.44 mg GAE/g), *H*. *aurantiaca* (30.80 mg GAE/g) and *H*. *paradoxa* (27.88 mg GAE/g), whereas several species like *D*. *confragosa*, *L*. *helvus*, *C*. *pyxidata*, *C*. *armillatus*, *R*. *maculate* and *T*. *terrestris* contained less than 5 mg GAE/g. The Folin–Ciocalteu’s method is commonly used for total phenolic content determination; however, the results obtained by different authors are difficult to compare due to different ways of expression. The study carried out by Karaman et al. by using a slightly modified method of Fukumoto and Mazza (2000) with the Folin–Ciocalteu reagent revealed that medicinal mushroom *F*. *fomentarius* contained 43.06 mg GAE/g of ethanolic extract [15, 16]. The study conducted by Paloi and Acharya (2013) showed that *A*. *vaginata* contained 5.335 μg GAE/mg of extract, which is a similar value to two species from the same family examined in our study—*A*. *pantherina* (5.27 mg GAE/g) and *A*. *muscaria* (7.59 mg GAE/g) [17]. Sułkowska-Ziaja et al. (2012) also investigated some mushrooms from Poland and obtained almost the same TPC value for *F*. *pinicola* (21.88 mg/g) as the one in our study (20.71 mg/g) [18]. The next study on chemical composition of Portuguese mushrooms disclosed that *L*. *vellereus* contained 12.62 mg GAE/g while in our analysis this species contained only 5.17 mg GAE/g [19]. The quantitative differences observed may be related to the place of harvesting and climatic conditions.

**Table 1:** Total phenolic content (TPC) and antiradical activity of mushroom extracts. TPC expressed as mg of gallic acid equivalents per gram of dried extract; IC_50_ expressed as mg of dry extract per mg DPPH^●^; AE—antiradical efficiency (1/IC_50_); TE—Trolox equivalent; VCE -ascorbic acid equivalent. Equivalents were calculated by dividing extract mean IC_50_ by standard mean IC_50_. For Trolox IC_50_ = 0.134 mg/mg DPPH^●^; for ascorbic acid IC_50_ = 0.146 mg/mg DPPH^●^. Mean values of three replicate assays with standard deviations.

Species	TPC	IC_50_	AE	TE	VCE
***Amanita citrina***	38.44±1.67	2.07±0.05	0.48	15.45	14.18
***Amanita muscaria***	7.59±0.25	18.1±0.4	0.06	135.08	123.97
***Amanita pantherina***	5.27±0.26	42.04±0.6	0.02	313.73	287.95
***Amanita porphyria***	14.53±0.54	5.07±0.03	0.20	37.84	34.73
***Bjerkandera adusta***	12.46±0.42	16.87±0.44	0.06	125.90	115.55
***Clavicorona pyxidata***	4.24±0.21	88.25±1.63	0.01	658.58	604.45
***Cortinarius armillatus***	4.50±0.10	28.18±0.65	0.04	210.30	193.014
***Cortinarius sanguineus***	8.79±0.38	16.78±0.29	0.06	125.22	114.93
***Daedaleopsis confragosa***	3.17±0.14	34.03±0.24	0.03	253.96	233.08
***Fomes fomentarius***	53.13±2.68	1.39±0.02	0.72	10.37	9.52
***Fomitopsis pinicola***	20.71±0.63	3.24±0.05	0.31	24.18	22.19
***Gymnopilus penetrans***	14.20±0.64	17.49±0.21	0.06	130.52	119.80
***Heterobasidion annosum***	12.70±0.55	5.27±0.07	0.19	39.33	36.10
***Hygrophoropsis aurantiaca***	30.80±1.27	4.44±0.13	0.23	33.13	30.41
***Hyphodontia paradoxa***	27.88±0.95	20.44±0.65	0.05	152.54	140.00
***Lactarius aurantiacus***	10.28±0.50	61.32±2.02	0.02	457.61	420.00
***Lactarius helvus***	4.10±0.13	82.42±1.6	0.01	615.08	564.52
***Lactarius vellereus***	5.17±0.25	113.37±2.61	0.01	846.05	776.51
***Lenzites betulinus***	7.46±0.22	18.41±0.04	0.05	137.39	126.10
***Panellus stypticus***	4.14±0.11	68.19±0.38	0.02	508.88	467.06
***Pseudoclitocybe cyanthiformis***	6.83±0.00	10.75±0.21	0.09	80.22	73.63
***Psilocybe fascicularis***	12.11±0.20	3.13±0.01	0.32	23.36	21.44
***Psilocybe lateritia***	5.66±0.12	20.5±0.35	0.05	152.99	140.41
***Rhodocollybia maculata***	4.87±0.18	60.67±0.28	0.02	452.76	415.55
***Russula fragilis***	6.00±0.02	120.54±2.46	0.01	899.55	825.62
***Scleroderma citrinum***	11.03±0.55	11.49±0.31	0.09	85.75	78.7
***Stereum hirsutum***	8.70±0.37	18.84±0.34	0.05	140.60	129.04
***Thelephora terrestris***	4.88±0.22	23.9±0.53	0.04	178.36	163.70
***Trametes hirsuta***	7.96±0.27	25.58±0.73	0.04	190.90	175.21
***Trichaptum fuscoviolaceum***	2.79±0.04	59.54±2.01	0.02	444.33	407.81
***Tubaria furfuracea***	4.30±0.18	74.15±0.18	0.01	553.36	507.88

### LC-ESI-MS/MS analysis of phenolic acids

Previous studies demonstrated the presence of hydroxybenzoic acid and hydroxycinnamic acid derivatives (mostly in the bound form, e.g. linked to sugars or to cell-wall structural components) in different mushroom species [20]. Therefore, qualitative and quantitative determination of 14 phenolic acids: gallic, protocatechuic, gentisic, 4-OH-benzoic, vanillic, caffeic, syringic, *p*-coumaric, ferulic, salicylic, veratric, synapic, 3-OH-cinnamic and rosmarinic in Polish mushrooms was conducted by the LC-ESI-MS/MS method. Conditions of LC-ESI-MS/MS analysis are given in Tables 2 and 3. The mushroom extracts analyzed in this research were found to contain both benzoic (protocatechuic, 4-OH-benzoic, vanillic, syringic) and cinnamic acid derivatives (caffeic, *p*-coumaric, ferulic) (Table 4). Our study disclosed the lack of gallic, gentisic, veratric, synapic, 3-OH-cinnamic and rosmarinic acid in the investigated fungi. *F*. *pinicola* showed the highest concentration of total phenolic acids (147.83 μg/g dry weight), mostly due to the contribution of protocatechuic acid (146.1 μg/g). The content of this acid was higher than value obtained in *F*. *pinicola* by Sułkowska-Ziaja et al. (2012) (114.9 μg/g dry weight) [18]. *G*. *penetrans* and *C*. *sanguineus* contained also quite high quantities of phenolic acids, 45.83 μg/g and 37.06 μg/g, respectively with the predominant 4-OH-benzoic acid. *G*. *penetrans* possessed also the highest amount of *p*-coumaric acid (9.04 μg/g). Generally, protocatechuic, 4-OH-benzoic and *p*-coumaric were the most typical for all 31 species analyzed. Salicylic acid occurred almost in all mushrooms but mostly in trace amounts, which is similar to the findings of our previous report concerning edible mushrooms [4]. Among the examined species, *H*. *paradoxa* contained the most diverse composition of phenolic acids and was characterized by the highest content of vanillic acid. No phenolic acids were detected in *L*. *vellereus* and *P*. *stypticus*, while *T*. *furfuracea* possessed only salicylic acid in trace amounts. This result differs from the data presented by Dogan and Aydin (2013) where ferulic, *p*-coumaric and cinnamic acid in *L*. *vellereus* were identified. However, their study was conducted on the material collected in Turkey and with the use of extraction with methanol at 60°C in the Soxhlet apparatus [21]. Extraction at elevated temperature might lead to partial release of bound polyphenolics, which would be consistent with the observations of Choi et al. (2006). In their study the heat treatment of *L*. *edodes* increased the total content of polyphenols [22]. According to the available data, the content of phenolic acids in *A*. *citrina*, *A*. *porphyria*, *B*. *adusta*, *C*. *pyxidata*, *C*. *sanguineus*, *G*. *penetrans*, *H*. *annosum*, *H*. *paradoxa*, *L*. *helvus*, *L*. *betulinus*, *P*. *stypticus*, *P*. *cyanthiformis*, *P*. *fascicularis*, *P*. *laterita*, *R*. *maculate*, *R*. *fragilis*, *S*. *citrinum*, *S*. *hirsutum*, *T*. *terrestris*, *T*. *fuscoviolaceum*, *T*. *furfuracea* was determined for the first time.

**Table 2:** LC-ESI-MS/MS analytical results of phenolic acids investigated in mushroom extracts, including retention times, molecular masses, mass-to-charge ratio (m/z) and fragments obtained with given collision energy. Compounds confirmed by comparison with authentic standards.

Compound	Peak no.	T_R_ (min)	M (g/mol)	m/z experimental	Fragments	Collision energy (eV)
**Gallic acid**	1	0.74	170.12	168.7	124.9	- 14
					78.9	- 36
**Protocatechuic acid**	2	1.70	154.12	152.9	107.8	- 38
					80.9	- 26
**Gentisic acid**	3	2.70	154.12	152.8	107.9	- 36
					81	- 30
**4-OH-benzoic acid**	4	3.26	138.12	136.8	92.9	- 18
**Vanillic acid**	5	4.49	168.15	166.8	107.9	- 18
					123	- 12
**Caffeic acid**	6	4.65	180.16	178.7	134.9	- 16
					88.9	- 46
**Syringic acid**	7	5.26	198.17	196.9	181.9	- 12
					122.8	- 24
**p-Coumaric acid**	8	5.60	164.16	162.7	119	- 14
					93	- 44
**Ferulic acid**	9	5.77	194.18	192.8	177.9	- 12
					133.9	- 16
**Salicylic acid**	10	5.80	138.12	136.8	93	- 16
					75	- 48
**Veratric acid**	11	5.80	182.17	180.7	136.9	- 12
					121.9	- 18
**Synapic acid**	12	5.81	224.21	222.8	148.9	- 20
					121	- 36
**3-OH-cinnamic acid**	13	5.82	164.16	162.8	119	- 14
					91	- 36
**Rosmarinic acid**	14	5.97	360.31	358.7	160.8	- 20
					132.6	- 44

**Table 3:** Analytical parameters of LC-ESI-MS/MS quantitative method; data for calibration curves, limit of detection (LOD) and limit of quantification (LOQ) values for each analyzed phenolic acid.

Compound	LOQ [ng/μl]	LOD [ng/μl]	*r* ^*2*^	Linearity range [ng/μl]
Gallic acid	0.10	0.05	0.9994	0.10–10
Protocatechuic acid	0.02	0.01	0.9991	0.025–3.13
Gentisic acid	0.015	0.008	0.9993	0.025–25
4-OH-benzoic acid	0.10	0.05	0.9971	0.10–2.5
Vanillic acid	0.20	0.10	0.9999	0.20–50
Caffeic acid	0.08	0.04	0.9972	0.08–1.25
Syringic acid	0.10	0.05	0.9997	0.10–50
p-Coumaric acid	0.061	0.018	0.9971	0.10–10.2
Ferulic acid	0.025	0.01	0.9997	0.025–5
Salicylic acid	0.02	0.01	0.9986	0.02–0.5
Veratric acid	0.70	0.40	0.9977	0.50–25
Synapic acid	0.025	0.007	0.9987	0.025–5
3-OH-cinnamic acid	0.05	0.02	0.9994	0.05–2.5
Rosmarinic acid	0.01	0.005	0.9985	0.025–25

**Table 4:** Phenolic acid contents in mushroom extracts expressed in μg per g of dry weight of mushrooms. Abbreviations: “-”not detected; Trace—trace amounts. Mean values of three replicate assays with standard deviation.

Species	Phenolic acids content [μg/g DW]								
	Protocatechuic	4-OH-benzoic	Vanillic	Caffeic	Syringic	*p*-coumaric	Ferulic	Salicylic	Sum
***Amanita citrina***	1.11±0.05	1.11±0.05	-	-	-	Trace	-	-	2.22
***Amanita muscaria***	-	-	-	-	-	-	-	0.66±0.06	0.66
***Amanita pantherina***	2.56±0.04	-	-	-	-	-	-	Trace	2.56
***Amanita porphyria***	0.2±0.01	0.47±0.01	-	-	-	-	-	-	0.67
***Bjerkandera adusta***	2.56±0.01	0.94±0.01	0.9±0.03	-	-	-	-	Trace	4.4
***Clavicorona pyxidata***	-	2.82±0.05	-	-	-	-	-	Trace	2.82
***Cortinarius armillatus***	0.13±0.0	-	-	-	-	0.02±0.0	-	-	0.15
***Cortinarius sanguineus***	-	35.55±0.1	-	-	-	1.51±0.02	-	-	37.06
***Daedaleopsis confragosa***	-	1.3±0.02	1.46±0.04		1.6±0.01	0.1±0.0	0.05±0.0	0.1±0.0	4.61
***Fomes fomentarius***	1.73±0.02	3.66±0.09	-	1.35±0.03	-	0.19±0.0	Trace	0.09±0.0	7.02
***Fomitopsis pinicola***	146.1±1.27	1.17±0.02	-	-	-	0.56±0.02	-	Trace	147.83
***Gymnopilus penetrans***	3.33±0.07	30.45±0.55	-	2.86±0.02	-	9.04±0.18		0.15±0.0	45.83
***Heterobasidion annosum***	-	5.04±0.02	-	-	-	-	-	-	5.04
***Hygrophoropsis aurantiaca***	2.0±0.06	4.19±0.05	-	-	-	0.4±0.01	-	Trace	6.59
***Hyphodontia paradoxa***	5.38±0.1	1.42±0.06	6.1±0.05	1.14±0.02	3.78±0.09	5.51±0.13	0.18±0.0	Trace	23.51
***Lactarius aurantiacus***	-	2.07±0.04	-	-	-	0.8±0.02	0.75±0.01	0.07±0.0	3.69
***Lactarius helvus***	0.79±0.01	2.41±0.04	-	-	-	0.44±0.0	-	Trace	3.64
***Lactarius vellereus***	-	-	-	-	-	-	-	-	-
***Lenzites betulinus***	0.67±0.0	-	1.43±0.04	0.16±0.01	1.36±0.06		0.16±0.0	0.10±0.0	3.88
***Panellus stypticus***	-	-	-	-	-	-	-	-	-
***Pseudoclitocybe cyanthiformis***	1.35±0.05	2.57±0.04	-	-	-	0.81±0.0	-	0.14±0.0	4.87
***Psilocybe fascicularis***	13.5±0.08	7.33±0.12	-	Trace	-	1.15±0.01	Trace	Trace	21.98
***Psilocybe lateritia***		19.57±0.01	-	-	-	-	-	-	19.57
***Rhodocollybia maculata***	16.75±0.21	-	-	-	-	-	-	0.11±0.0	16.86
***Russula fragilis***	0.15±0.0	-	-	-	-	-	-	Trace	0.15
***Scleroderma citrinum***	0.41±0.02	5.43±0.11	-	-	-	-	-	-	5.84
***Stereum hirsutum***	-	0.74±0.02	-	-	-	0.2±0.0	-	0.03±0.0	0.97
***Thelephora terrestris***	2.48±0.12	2.94±0.03	-	-	18.77±0.15	1.21±0.05	Trace	0.12±0.0	25.52
***Trametes hirsuta***	0.87±0.08	0.98±0.01	-	-	-	0.08±0.0		Trace	1.93
***Trichaptum fuscoviolaceum***	1.89±0.06	0.37±0.01	0.75±0.02	-	-	0.03±0.0	0.04±0.0	Trace	3.08
***Tubaria furfuracea***	-	-	-	-	-	-	-	Trace	-

### DPPH^●^ assay

Numerous wild growing mushrooms possess significant antioxidant potential which is often related to the phenolic compound content. There are several protocols to determine this activity [4, 20]. Therefore we wanted to conduct broad analysis of antiradical activity of Polish mushroom extracts against DPPH^●^ free radical. The IC_50_ values and results expressed as antiradical efficiency, Trolox and ascorbic acid equivalents are presented in Table 1. The dose response curves and r^2^ values for each extract are listed in S1 Table. Generally, all extracts can be divided into three groups according to their antiradical potential. The first group with IC_50_ values < 10, thus highly active, contains seven species: *A*. *citrina*, *A*. *porphyria*, *F*. *fomentarius*, *F*. *pinicola*, *H*. *annosum*, *H*. *aurantiaca* and *P*. *fascicularis*. The activity of this group is comparable with antioxidant potential of effective radical scavengers of plant origin [13]. The second group showing moderate activity (IC_50_ from 10 to 50) consists of fifteen species, and is quite diverse in terms of taxonomic affiliation. The third group possessing low activity (IC_50_ > 50) contains nine species, including four species representing the *Russulaceae* family (i.e. *L*. *aurantiacus*, *L*. *helvus*, *L*. *vellereus*, *R*. *fragilis*). We have also paid closer attention to whether mushrooms living on trees or on the remains of dead trees demonstrate different activity than those growing in soil. The findings did not disclose very significant differences. Saprophytic polyphores were found to exhibit various antiradical activities. However, all investigated parasitic fungi (*F*. *fomentarius*, *F*. *pinicola* and *H*. *annosum*) from living trees revealed very strong antioxidant potential with IC_50_ value below 10 mg/mg DPPH^●^. As far as we know, this is the first report revealing antiradical activity of *A*. *citrina*, *A*. *porphyria*, *B*. *adusta*, *C*. *pyxidata*, *G*. *penetrans*, *H*. *annosum*, *H*. *paradoxa*, *P*. *stypticus*, *P*. *cyanthiformis*, *P*. *fascicularis*, *P*. *lateritia*, *R*. *maculate*, *R*. *fragilis*, *T*. *terrestris*, *T*. *fuscoviolaceum* and *T*. *furfuracea*. Several previous reports demonstrated a significant relationship between total phenolic content and antioxidant activity [23, 24]. In our study we observed such a correlation in *F*. *fomentarius*, which possessed the strongest antiradical activity (IC_50_ 1.39 mg/mg DPPH^●^) and, as it was mentioned before, the highest TPC. *A*. *citrina*, *P*. *fascicularis* and *F*. *pinicola* demonstrated relatively high scavenging activity, whereas *R*. *fragilis* was found to be the least active (IC_50_ 120.54 mg/mg DPPH^●^). In general, the correlation coefficient between antioxidant activity (IC_50_) and total phenolic content (TPC) for all analyzed mushrooms amounted to -0.496, which indicates quite significant dependence. It can be related to the presence of other groups of compounds with antioxidant activity in mushrooms, e.g. tocopherols, ascorbic acid and carotenoids [20].

### Antibacterial assay *in vitro*

The final aim of this research was to determine the antimicrobial potential of 31 mushroom extracts. We investigated antibacterial activity against Gram-positive (*S*. *epidermidis*, *S*. *aureus*, *B*. *subtilis*, *M*. *luteus*) and Gram-negative (*E*. *coli*, *K*. *pneumoniae*, *P*. *aeruginosa*, *P*. *mirabilis*) microbial strains. The results are presented in Table 5. *H*. *paradoxa* was found to be the most active mushroom against all Gram-positive bacteria, with minimal inhibitory concentration values ranging from 0.156 to 0.625 mg/ml. On the other hand, *F*. *pinicola* demonstrated strong activity against Gram-negative bacteria, with MIC values from 0.625 to 2.5 mg/ml. Generally, slightly higher activities of mushrooms against Gram-positive than Gram-negative strains were observed, which is comparable with our previous results regarding antimicrobial activity of edible mushrooms [4]. *M*. *luteus*, *B*. *subtilis* and *P*. *aeruginosa* were the most susceptible strains to the inhibitory effect of mushrooms, while *P*. *mirabilis* was the most resistant. For every extract examined, we also determined the minimal bactericidal concentration (MBC), which was subsequently compared with the corresponding MIC value. The MBC to MIC ratio indicates bactericidal properties when it ranges from 1 to 4. Almost all examined fungus species demonstrated this activity. To the best of our knowledge, this is the first screening on antimicrobial activities of these 31 mushroom species from Poland.

**Table 5:** Antimicrobial activity of mushroom extracts expressed in mg of dry extract per ml. Abbreviations: R—MBC/MIC ratio; “-”not determined; MICs of gentamicin ranged from 0.03–0.12 x 10–3 mg/ml and 0.25–1.0 x 10–3 mg/ml for Gram-positive and Gram-negative bacterial strains, respectively; DMSO at the final concentration used had no influence on the growth of strains. Mean values of three replicate assays.

Species	Reference microbial strains																							
	Gram-positive bacteria												Gram-negative bacteria											
	*S*. *epidermidis*			*S*. *aureus*			*B*. *subtilis*			*M*. *luteus*			*E*. *coli*			*K*. *pneumoniae*			*P*. *areuginosa*			*P*. *mirabilis*		
	ATCC 12228			ATCC 25923			ATCC 6633			ATCC 10240			ATCC 25922			ATCC 13883			ATCC 9027			ATCC 12453		
	MIC	MBC	R	MIC	MBC	R	MIC	MBC	R	MIC	MBC	R	MIC	MBC	R	MIC	MBC	R	MIC	MBC	R	MIC	MBC	R
***Amanita citrina***	2.5	2.5	1	5	5	1	5	5	1	2.5	5	2	2.5	2.5	1	2.5	2.5	1	0.625	1.25	2	2.5	5	2
***Amanita muscaria***	2.5	5	2	2.5	5	2	1.25	2.5	2	0.625	2.5	4	2.5	>5	-	2.5	>5	-	1.25	2.5	2	2.5	>5	-
***Amanita pantherina***	1.25	2.5	2	2.5	5	2	0.625	1.25	2	0.625	1.25	2	1.25	2.5	2	2.5	2.5	1	0.625	1.25	2	2.5	5	2
***Amanita porphyria***	2.5	2.5	1	2.5	5	2	2.5	2.5	1	2.5	2.5	1	1.25	1.25	1	2.5	2.5	1	1.25	1.25	1	2.5	5	2
***Bjerkandera adusta***	2.5	5	2	2.5	5	2	1.25	5	4	1.25	2.5	2	2.5	5	2	2.5	>5	-	1.25	1.25	1	2.5	5	2
***Clavicorona pyxidata***	2.5	2.5	1	2.5	5	2	1.25	1.25	1	1.25	2.5	2	2.5	2.5	1	2.5	5	2	0.625	1.25	2	2.5	5	2
***Cortinarius armillatus***	5	5	1	>5	>5	-	1.25	2.5	2	2.5	5	2	1.25	2.5	2	1.25	2.5	2	1.25	2.5	2	2.5	5	2
***Cortinarius sanguineus***	2.5	5	2	5	>5	-	2.5	2.5	1	1.25	5	4	2.5	2.5	1	1.25	2.5	2	1.25	2.5	2	2.5	5	2
***Daedaleopsis confragosa***	5	5	1	5	>5	-	5	5	1	1.25	5	4	2.5	5	2	5	>5	-	2.5	5	2	2.5	>5	-
***Fomes fomentarius***	1.25	2.5	2	1.25	5	4	1.25	2.5	2	1.25	2.5	2	2.5	2.5	1	1.25	2.5	2	1.25	1.25	1	1.25	2.5	2
***Fomitopsis pinicola***	2.5	5	2	5	>5	-	2.5	5	2	1.25	2.5	2	0.625	0.625	1	2.5	5	2	0.625	1.25	2	2.5	5	2
***Gymnopilus penetrans***	2.5	2.5	1	5	>5	-	1.25	2.5	2	0.625	2.5	4	2.5	2.5	1	2.5	>5	-	2.5	5	2	2.5	5	2
***Heterobasidion annosum***	1.25	1.25	1	1.25	2.5	2	<0.625	0.625	-	1.25	5	4	1.25	1.25	1	1.25	1.25	1	<0.625	0.625	-	2.5	5	2
***Hygrophoropsis aurantiaca***	2.5	2.5	1	2.5	5	2	2.5	2.5	1	2.5	2.5	1	2.5	2.5	1	2.5	>5	-	2.5	5	2	2.5	5	2
***Hyphodontia paradoxa***	0.625	1.25	2	0.625	0.625	1	0.313	0.625	2	0.156	0.625	4	1.25	2.5	2	2.5	5	2	2.5	5	2	2.5	5	2
***Lactarius aurantiacus***	2.5	5	2	2.5	5	2	1.25	5	4	1.25	1.25	1	2.5	2.5	1	2.5	>5	-	2.5	5	2	2.5	5	2
***Lactarius helvus***	2.5	5	2	2.5	>5	-	1.25	2.5	2	1.25	1.25	1	2.5	2.5	1	2.5	5	2	1.25	2.5	2	2.5	5	2
***Lactarius vellereus***	5	5	1	5	>5	-	1.25	1.25	1	1.25	1.25	1	2.5	>5	-	2.5	>5	-	1.25	1.25	1	2.5	>5	-
***Lenzites betulinus***	1.25	2.5	2	1.25	2.5	2	1.25	5	4	1.25	2.5	2	2.5	2.5	1	2.5	2.5	1	2.5	2.5	1	2.5	>5	-
***Panellus stypticus***	2.5	2.5	1	2.5	5	2	2.5	5	2	2.5	2.5	1	2.5	2.5	1	2.5	>5	-	2.5	2.5	1	2.5	5	2
***Pseudoclitocybe cyanthiformis***	2.5	2.5	1	2.5	5	2	2.5	5	2	2.5	2.5	1	2.5	5	2	2.5	5	2	2.5	5	2	2.5	5	2
***Psilocybe fascicularis***	2.5	5	2	2.5	>5	-	1.25	2.5	2	1.25	2.5	2	2.5	2.5	1	2.5	>5	-	2.5	5	2	2.5	5	2
***Psilocybe lateritia***	2.5	5	2	2.5	>5	-	2.5	5	2	1.25	1.25	1	2.5	5	2	2.5	>5	-	2.5	5	2	2.5	>5	-
***Rhodocollybia maculata***	5	5	1	5	5	1	5	5	1	2.5	5	2	2.5	2.5	1	5	>5	-	2.5	5	2	2.5	5	2
***Russula fragilis***	5	5	1	5	5	1	2.5	2.5	1	1.25	2.5	2	2.5	>5	-	2.5	>5	-	1.25	1.25	1	2.5	>5	-
***Scleroderma citrinum***	2.5	5	2	5	>5	-	1.25	2.5	2	1.25	2.5	2	2.5	5	2	1.25	5	4	1.25	1.25	1	2.5	>5	-
***Stereum hirsutum***	2.5	2.5	1	2.5	2.5	1	2.5	5	2	2.5	2.5	1	2.5	2.5	1	2.5	2.5	1	2.5	2.5	1	5	5	1
***Thelephora terrestris***	2.5	2.5	1	2.5	2.5	1	2.5	5	2	2.5	2.5	1	2.5	2.5	1	2.5	2.5	1	2.5	5	2	2.5	5	2
***Trametes hirsuta***	2.5	2.5	1	2.5	2.5	1	2.5	2.5	1	2.5	2.5	1	2.5	2.5	1	2.5	2.5	1	2.5	2.5	1	2.5	5	2
***Trichaptum fuscoviolaceum***	2.5	2.5	1	2.5	2.5	1	1.25	>5	-	1.25	2.5	2	2.5	>5	-	2.5	5	2	2.5	2.5	1	2.5	5	2
***Tubaria furfuracea***	2.5	5	2	2.5	5	2	2.5	5	2	1.25	1.25	1	2.5	5	2	5	>5	-	1.25	1.25	1	2.5	>5	-

## Conclusions

As mentioned earlier, the wild growing fungi are still a poorly explored source of natural compounds. Since Poland is one of the European regions with relatively high wild mushroom diversity, there is a lot to be done in the area of their investigations. It is also important to characterize chemical composition and biological activity of different species particularly these which could be obtained in industrial amounts. Thus, the aim of this article was determination of chemical composition and biological activity of 31 wild growing fungi from Poland. As a result, the first such broad report on polyphenolic profile, antiradical and antimicrobial potential of wild growing Polish mushrooms is presented. The amounts of polyphenols were found to be even higher than quantities previously evaluated in edible mushrooms. Moreover, the presence of eight phenolic acids in the fruiting bodies of the tested species was revealed. It was also found that mushrooms constitute a good source of natural antioxidant and antimicrobial agents. Some species present antiradical capacity comparable to highly active plant extracts. Interestingly, three of them represent the group of parasitic fungi. Our screening study provides a wealth of information on the kingdom of fungi in Poland. We hope that it will encourage further exploration since more research is needed to effectively use natural forest resources.

## Supporting Information

S1 Table The dose response curves and r2 values used to determine the IC50 values for each extract. (DOCX)
