# Supplementary material for: Antibacterial, Antiradical Potential and Phenolic Compounds of Thirty-One Polish Mushrooms
Source: PLoS One. 2015 Oct 15;10(10):e0140355. doi: 10.1371/journal.pone.0140355 (PMC4607371; doi:10.1371/journal.pone.0140355)
Supplement: S1 Table — (DOCX) [file pone.0140355.s001.docx]

**S1 Table.** The dose response curves and r^2^ values used to determine the IC_50_ values for each extract.

| **Species** | **Dose response curves** | **r^2^** |  |  |  |
| --- | --- | --- | --- | --- | --- |
| ***Amanita citrina*** | y=1.7576x+4.0859 | 0.9976 |  |  |  |
| ***Amanita muscaria*** | y=0.2024x+3.8328 | 0.9978 |  |  |  |
| ***Amanita pantherina*** | y=0.0912x+1.6517 | 0.9951 |  |  |  |
| ***Amanita porphyria*** | y=0.7022x+4.2878 | 0.9941 |  |  |  |
| ***Bjerkandera adusta*** | y=0.1911x+9.3427 | 0.9962 |  |  |  |
| ***Clavicorona pyxidata*** | y=0.0266x+20.4687 | 0.9921 |  |  |  |
| ***Cortinarius armillatus*** | y=0.1301x+3.7735 | 0.9988 |  |  |  |
| ***Cortinarius sanguineus*** | y=17.89x+12.1753 | 0.9922 |  |  |  |
| ***Daedaleopsis confragosa*** | y=0.1088x+3.3237 | 0.9945 |  |  |  |
| ***Fomes fomentarius*** | y=2.3592x+8.8345 | 0.9957 |  |  |  |
| ***Fomitopsis pinicola*** | y=1.0397x+7.5456 | 0.9988 |  |  |  |
| ***Gymnopilus penetrans*** | y=0.1630x+14.0545 | 0.9952 |  |  |  |
| ***Heterobasidion annosum*** | y=1.3285x-38.4879 | 0.9939 |  |  |  |
| ***Hygrophoropsis aurantiaca*** | y=0.2826x+34.1818 | 0.9925 |  |  |  |
| ***Hyphodontia paradoxa*** | y=0.1361x+14.9151 | 0.9972 |  |  |  |
| ***Lactarius aurantiacus*** | y=0.368x+21.4920 | 0.9957 |  |  |  |
| ***Lactarius helvus*** | y=0.341x+14.6341 | 0.9914 |  |  |  |
| ***Lactarius vellereus*** | y=0.0321x+4.2297 | 0.9961 |  |  |  |
| ***Lenzites betulinus*** | y=0.1959x+4.5549 | 0.9967 |  |  |  |
| ***Panellus stypticus*** | y=0.0327x+21.9379 | 0.9950 |  |  |  |
| ***Pseudoclitocybe cyanthiformis*** | y=0.3051x+8.6998 | 0.9972 |  |  |  |
| ***Psilocybe fascicularis*** | y=1.1394x+5.0059 | 0.9966 |  |  |  |
| ***Psilocybe lateritia*** | y=0.1916x+0.5226 | 0.9997 |  |  |  |
| ***Rhodocollybia maculata*** | y=0.0373x+21.4826 | 0.9927 |  |  |  |
| ***Russula fragilis*** | y=0.0261x+10.4885 | 0.9951 |  |  |  |
| ***Scleroderma citrinum*** | y=0.3403x+0.9354 | 0.9962 |  |  |  |
| ***Stereum hirsutum*** | y=0.1705x+9.5209 | 0.9981 |  |  |  |
| ***Thelephora terrestris*** | y=0.1334x+9.8260 | 0.9940 |  |  |  |
| ***Trametes hirsuta*** | y=0.1238+10.1031 | 0.9989 |  |  |  |
| ***Trichaptum fuscoviolaceum*** | y=0.0175x+36.8612 | 0.9961 |  |  |  |
| ***Tubaria furfuracea*** | y=0.0310x+21.0093 | 0.9957 |  |  |  |
